# Bioceramic cement in the filling of bone defects in rats^1^

**DOI:** 10.1590/s0102-865020190060000001

**Published:** 2019-08-19

**Authors:** Christiano Cândido Zerbinatti, Daniela Francescato Veiga, Monique Amanda Bastista Oliveira, Fiorita Gonzales Lopes Mundim, Rodrigo Machado Pereira, Francisco Azevedo, Taylor Brandão Schnaider, José Dias da Silva

**Affiliations:** I DDS, Master, Professional Masters in Sciences Applied to Health, Universidade Vale do Sapucaí (UNIVÁS), Pouso Alegre-MG, Brazil. Conception and design of the study; acquisition, analysis and interpretation of data; manuscript preparation.; II PhD, Professional Masters in Sciences Applied to Health, UNIVÁS, Pouso Alegre-MG, Brazil. Critical revision, final approval.; III Graduate student, Biological Sciences, UNIVÁS, Pouso Alegre-MG, Brazil. Technical procedures.; IV PhD, Professional Masters in Sciences Applied to Health, UNIVÁS, Pouso Alegre-MG, Brazil. Histopathological examinations.; V Master, Biological Sciences, UNIVÁS, Pouso Alegre-MG, Brazil.Ttechnical procedures, histological examinations, manuscript writing.; VI DDS, Master, Professional Masters in Sciences Applied to Health, UNIVÁS, Pouso Alegre-MG, Brazil. Technical procedures, statistics analysis.; VII PhD, Professional Masters in Sciences Applied to Health, UNIVÁS, Pouso Alegre-MG, Brazil. Technical procedures, statistics analysis, critical revision.; VIII PhD, Professional Masters in Sciences Applied to Health, UNIVÁS, Pouso Alegre-MG, Brazil. Scientific and intellectual content of the study, analysis and interpretation of data, manuscript writing, critical revision, final approval.

**Keywords:** Dental Implants, Immediate Dental Implant Loading, Guided Tissue Regeneration, Periodontal, Bone Substitutes, Rats

## Abstract

**Purpose:**

To evaluate PBS^®^MCIMMO cement in the filling of bone defects.

**Methods:**

Thirty-six adult male Wistar rats were divided into three groups of twelve individuals each (group 1, group 2 and group 3). In all groups, a bone failure in the femur was induced, 2.0 mm wide and 7.0 mm deep. In group 1, the PBS^®^MCIMMO cement was applied to the bone defect produced and a titanium implant (CONNECTION^®^) 1.5 mm thick and 6 mm long was installed. In group 2, only the PBS^®^ CIMMO cement was installed. In group 3, only bone failure was performed. Kruskal Wallis tests were performed to compare the mean area among the three groups.

**Results:**

In all comparisons, significance was observed for group 2 (p = 0.0014–0.0026).

**Conclusion:**

The PBS^®^CIMMO cement induced bone neoformation, and integration between the newly formed bone, cement, and implant was observed.

## Introduction

The exodontia of anterior dental elements induces bone failure requiring immediate aesthetic reconstruction through the installation of implants and the preparation of provisional or definitive prosthesis. Autologous, homologous or allogeneic bone grafts do not allow immediate procedures. Using biomaterials to preserve the architecture of the alveolus - height and width - would allow immediate aesthetic reconstruction^1-3^.

The fresh alveoli of newly extracted dental elements, without immediate functional restorative management of the lost element, will undergo remodeling during the healing process, resulting in a reduction of the alveolar margin inducing bone defects leading to serious difficulties in the reconstruction and rehabilitation through dental implants^1-4^.

Immediate implantation is the best and fastest way to preserve the alveolar margin and to rehabilitate the patient. However, the indication for this treatment modality depends on several factors, including adequate bone support, presence of buccal bone table, and favorable gingival phenotype^5^. One of the problems of implant dentistry is related to the treatment of patients whose alveolar remnant does not meet the requirements for immediate implantation, i.e. primary stability, and the juxtaposition of the implant to the walls of the surgical alveolus^6^.

Implant installations are not possible when the alveolar bone does not provide primary stability due to spaces lying between the surface of the implant and the bone board^7^. The reconstruction of these alveoli is usually performed through a bone graft. However, these procedures do not increase the primary stability for immediate implants^8,9^.

The advent of bioactive cements (BACS) marked a new era in dentistry, mainly due to the possibility of rehabilitating dental roots condemned by persistent infections and perforations^14-15^. Biomaterials with osteoinductive characteristics, defined by bioactivity and the addition of cations, could solve the primary stability problem for immediate implants^11-14^. We propose an experimental study using bioactive cement to fill experimental bone defects, aiming at testing the possibility of immediate implants.

## Methods

The project was submitted to the Committee on Ethics in the Use of Animals (CEUA) and approved under opinion 265/17.

This study is a primary, longitudinal, prospective, and analytical experimental research. Thirty-six male Wistar rats with a mean weight of 350 to 450g were kept in a controlled temperature environment (22 ± 2ºC), with a light / dark cycle of 12 h (light after 7:00 am) and water *ad libitum* in the laboratory of Universidade Vale do Sapucaí (UNIVÁS), after completion of the quarantine period. The animals were kept in individual cages and fed balanced commercial feed. In the week preceding the experimental phase, clinical examination and care were performed by a veterinarian. The animals had normal health and activity during the quarantine period. Rats that died during the experiment were excluded from the analysis.

The animals were divided into 3 groups of 12 rats each, according to statistical planning^15^, and surgical technique planning was performed^16-18^.

Benzothiazine benzene (600.000 IU), benzylpenicillin procaine (300.000 IU), benzylpenicillin potassium (300.000 IU), dihydrostreptomycin sulfate (250 mg), and streptomycin sulphate (250 mg) were used for antibiotic prophylaxis. Pentabiotic therapy (antibiotics for small animals) was administered orally at a dose of 1 mg / kg body weight every 12 hours, starting 24 hours before the surgical procedure, and then maintained for 7 days.

Sedation was performed with ketamine hydrochloride (70 mg/kg) (Ketalar^®^ 10%, Pfizer, São Paulo) and xylazine hydrochloride (6 mg/kg) (Rompum^®^ 2% - Bayer SA) intramuscularly. Trichotomy was performed in the femoral region with a razor (GILLETE^®^) followed by local infiltrative anesthesia (12.5 mg/kg) of bupivacaine (Neocaína^®^ 5% - Cristália). The animals maintained spontaneous breathing.

Asepsis of the hands, forearms and clothing of the surgical team were ensured with a disposable, sterile TNT kit (Suprimed^®^). Skin disinfection was performed with chlorhexidine 2% (Chlorhexidine Diclonato - Riohex).

The same surgical procedure was performed in all rats, except for the specificities of each group. After anesthetic infiltration, tricotomy was performed and access to the femur was obtained through a cutaneous linear incision measuring 3 cm in length performed with a scalpel blade (Swann-Morton^®^)^15^. The flap was removed with a Molt 2/4 elevator and blunt scissors to expose the femur. A defect was introduced simulating a bone defect analogous to a dental socket in the proximal area of the femur surface: A hole with a maximum diameter of 2.0 mm in width and 7 mm in depth was drilled at a 45-degree angle with respect to the surface of the femur, through a reduction angle of 20:1, with an electric motor programmed at 1200 RPM (KAVO^®^) and constant irrigation with saline solution.

In group 1, PBS^®^ CIMMO cement mixed with distilled water was handled with a flexible spatula on a sterile glass plate, according to the manufacturer’s recommendations, and inserted into the defect. Before the cement solidified, an orthodontic anchorage implant (CONNECTION^®^), 1.5 mm thick and 6 mm long, was installed, using a manual installation wrench (CONNECTION^®^).

In group 2, the PBS^®^MCIMMO cement mixed with distilled water was handled with a flexible spatula on a sterile glass plate, according to the manufacturer’s recommendations, and inserted into the defect.

In group 3 (control), only femur drilling was performed.

Then, the periosteum was repositioned and an internal suture was performed by single dots with polyglactin 910c (Vicryl^®^ 4.0) J & J. The external suture of the dermis was performed with mononylon (4-0) - (SHALON^®^).

After 8 weeks of confinement, the rats were anesthetized with intramuscular injection of Ketamine Hydrochloride (70 mg/kg) and the femurs removed with bone dissection. The animals were euthanized with an intracardiac injection of Potassium Chloride (19.1% at 2 ml / kg).

The pieces were identified, packed in plastic containers containing 10% formalin and phosphate buffer (pH 7.2), and prepared for histological processing. Decalcification was performed in 10% EDTA solution for 15 days. The blocks were obtained by transverse sections with a thickness of 4 micrometers in a rotating microtome, resulting in semi-serial cuts that were subjected to staining by hematoxylin and eosin.

The structures were quantified by histological analysis. The injured region was evaluated by optical microscopy in order to evaluate the presence of repair and / or inflammation reaction processes. The presence of the following cellular and tissue constituents was also considered: connective tissue, fibroblasts, osteoblasts, osteoclasts and osteoid

For morphometry, images were captured with the Moticam M1000 digital camera, coupled to the optical microscope, using Motic Images Plus software. The images were captured at a magnification of x100, and exported in Bitmap format. For each sample, three distinct regions were selected for photomicrographs.

Repair areas were evaluated by manual measurement using ImageJ software version 1.51 (National Institute of Health, USA). To do so, the files were imported into the software. The lesion filling tissue was selected using the Color threshold function. The color, saturation and brightness ranges were adjusted so that the largest tissue area was selected, excluding unfilled areas. The area values were obtained by the Measure function and submitted to statistical analysis.

Group 1 samples were subjected to the histological processing technique in historesin preliminary cuts, followed by wear. Sections were stained with Stevenel’s blue in order to identify calcified bone tissue and to evaluate the tissue/cement/implant interaction according to Tian *et al*.^24^.

The Kruskal-Wallis test was performed. The significance level was set at 5% in all tests. Statistical analysis was performed with SPSS software.

## Results

The histomorphometry data of bone neoformation in groups 1, 2, and 3 were analyzed by measuring three areas (A, B and C) in the photomicrographs. The Kruskal Wallis test was performed to compare the mean area among the three groups. In all comparisons group 2 was significantly different from the other groups (p = 0.0014–0.0026, Table 1).

**Table 1:** Results of the analysis of groups 1, 2, and 3 in areas A, B, and C (Kruskal Wallis test).

	Areas		

	A	B	C
**H**	13.1655	11.9324	12.01006
**DF**	2	2	2
**P-value**	0.0014	0.0026	0.0024

**CT**	280.5000 μm	285.0000 μm	289.0000 μm
**CI**	271.5000 μm	261.0000 μm	257.0000 μm
**C**	114.0000 μm	120.0000 μm	120.0000 μm

**CT (Medium Post)**	23.3750 μm	23.7500 μm	24.0833 μm
**CI (Medium Post)**	22.6250 μm	21.7500 μm	21.4167 μm
**C (Medium Post)**	9.5000 μm	10.0000 μm	10.0000 μm

H = Kruskal – Wallis chi-squared; DF – Degrees of freedom; P-values are obtained with the Kruskal-Wallis test. CT – group 1; CI –group 2; C – group 3 (control).

**Figure 1:**
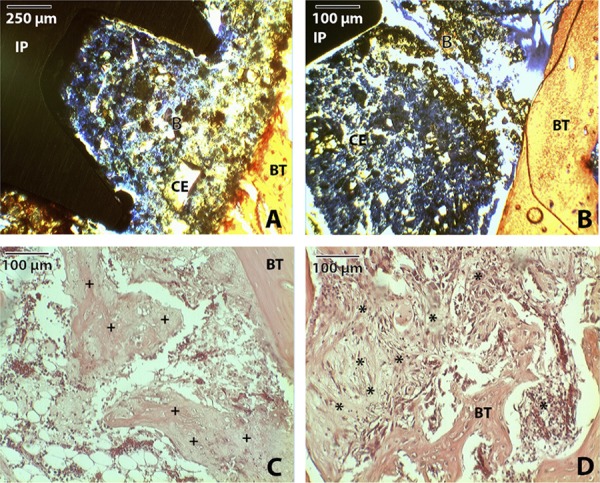
Newly formed cells. (A, B): histological profile of the defect with PBS cement. The integration between the implant (IP), the cement (CE), the bone tissue (BT) can be observed, and bone that formed inside the cement can be seen. Blue staining was obtained with the method of Stevenel and Alizarina Vermelha. (C, D): without implant, the presence of osteoid formation (+) and connective repair tissue (*) can be observed by hematoxylin and eosin staining.

The samples presented newly formed cells: Connective tissue, fibroblasts and osteoid cells were observed (Fig. 1A). Figure 1B shows the presence of mineralized bone tissue and connective tissue encased in the cement.

## Discussion

Exodontia is indicated in the following situations: trauma causing irreversible root fractures, secondary endodontic infections, and periodontal diseases that resist conventional treatments. The practitioner who performs the rehabilitation will face the problem of changes in bone continuity - bone defects - or segmental bone loss preventing immediate implantation^18^. A constant problem in the clinical practice of implant dentistry is the indication of extraction of dental elements in aesthetically relevant regions. In most cases, the immediate aesthetic reconstruction with implants and the preparation of provisional or definitive prosthesis is essential. However, bone failures need to be regenerated prior to implant installation that will replace the dental root, due to the lack of bone structure to support the implant. This process, called guided bone regeneration (GBR) requires time, when conventional techniques such as autologous, homologous, or allogeneic bone grafts are used^7,12,20^and thus aesthetics will be compromised^1^.

Alveolar reconstruction is best performed at the time of exodontia. When using biomaterials to preserve the architecture of the alveolus - height and width - better results will be obtained in rehabilitation with implants which can be installed immediately after the extraction, with immediate aesthetic rehabilitation^2,3^while achieving primary stability: Juxtaposition of the implant to the walls of the alveolus, mechanical stability, solidity, rigidity, and resistance to the movement of the implant, obtained at the moment of insertion^6^ can occur only when the alveolus does not present bone failure.

This study presented a proposal for the development of a protocol for the solution of the aforementioned problem, through the use of biomaterials for immediate reconstruction of the alveolus, allowing the installation of the implant. However, the implant is not juxtaposed directly to the walls of the bone cavity, but to the previously installed PBS^®^CIMMO cement. In the 12 specimens of group 1 mechanical stability, stiffness and resistance to movement of the implant occurred immediately upon installation. In addition to the mechanical results at the time of the experiment, histology demonstrated, in group 1, that after 8 weeks bone structures were entangled with cement and the implant, supporting the hypothesis that a new modality of integration has emerged in this study, namely implant-cement-bone integration.

The ideal biomaterial needs to have osteoinductive properties, defined in the literature by the term “bioactivity,” meaning interaction of its components with the bone, determining the production of carbonated apatites and newly formed bone. This property is related to biocompatibility, defined as the absence of toxicity and the compatibility with organic tissues^10,22^.

Among biomaterials, biological cements are of paramount importance because they allow solving previously impossible cases. These cements are based on the Portland cement used in civil construction, whose raw material is limestone^10,22^. Recent studies have allowed the development of biological cement (PBS^®^CIMMO rapid set) with the addition of additives (natural elements responsible for the increase of cement strength as well as the setting time) and without radioactivity^12^. The experimental and clinical studies that culminated in the development of PBS^®^CIMMO rapid set were designed for installation in crowns and dental root structures in situations called unconventional, because no protocols had been developed for these applications, and the roots were condemned^10-14,23^. However, recently, a study was carried out using PBS^®^CIMMO cement in a successful endodontic retreatment of alveolar abscesses^11^. The results of these studies, which demonstrated the total compatibility of the cement with the alveolar bone, stimulated the ideation of the present study.

An experimental model in which the cement was installed directly into the bone cavity was thus developed. The purpose dividing the samples into 3 groups was to provide a way to demonstrate that the cement, in contact with the alveolar bone alone, would induce neoformation. Group 2 (perforation and cement) indeed showed significant differences (p = 0.0014–0.0026) compared with controls. Together with previous reports, the present study confirms that the cement, placed into contact with the bone, promotes new bone formation.

The comparison between the groups showed no significant differences between groups 1 and 2 in terms of new bone formation, implying that new formation of differentiated bone is present in group 1, due to the presence of the implant, the connective tissues, and the bone and the formation of tangles between the cement and the implant. Regarding group 3, no new bone formation was observed, while inflammatory infiltrate was present. These findings indicate the bioactivity of the rapid set PBS^®^CIMMO cement.

It is relevant to report in this discussion the recovery of functionality and healing that occurred during the 8 weeks of confinement. Thirty-six Wistar rats were submitted to the experiment and a loss occurred at the end of 7 weeks. Number 5 specimen in group 1 developed diarrhea and was treated by the veterinarian responsible for the vivarium, but did not survive. According to the veterinarian’s report, diarrhea was unrelated to the experimental procedure, since it occurred 7 weeks after surgery, and throughout the postoperative period and until the seventh week the animal was in normal state, like the other thirty-five rats. We chose not to include this sample in the histological and histomorphometric analysis. Even so, it was observed that, functionally, the 36 rats behaved naturally during the confinement period, moving around and feeding naturally. Regarding healing, all rats presented tissue regeneration one week after the experiment, except specimens 35 and 33, in which, after 2 weeks, a small abscess formation was observed in the operated region. It is important to emphasize that these 2 rats were part of the control group (group 3), i.e. without cement. In the rats of groups 1 and 2 there were no signs or symptoms of altered health, corroborating the histological results.

The results of the present study indicate the possibility of using PBS^®^CIMMO fast-set cement in future clinical studies, extrapolating the present model and clinical protocol. This cement could become a material for synthetic grafting in fresh alveoli with immediate installation of implants and prostheses.

The PBS^®^ CIMMO cement allows simple and fast application with a reproducible technique. It does not require specialized training for its manipulation and use in routine surgical procedures. The tissue response and the possibility of increasing the primary stability of the implants, when used in immediate loading procedures in implant dentistry, can lead to the solution of some of the challenges faced by professionals in their daily practice. Obtaining stability after immediate loading is a desirable situation in implantology, and a model that increases the primary stability of the implants represents an important predictability factor for implantodontists.

## Conclusion

The cement PBS^®^CIMMO induced bone neoformation and integration was observed involving the newly formed bone, the cement, and the titanium screw.

## Footnotes

1 Research performed at the Laboratory of Bases of Surgical Techniques, Faculty of Medicine, and the Laboratory of Biology, Faculty of Biology, Universidade Vale do Sapucaí (UNIVÁS), Pouso Alegre-MG, Brazil. Part of Master’s degree thesis, Professional Masters in Sciences Applied to Health. Tutor: Prof. José Dias da Silva Neto.
